# Editorial: *Leishmania* genome variability: Impacts on parasite evolution, parasitism and leishmaniases control

**DOI:** 10.3389/fcimb.2023.1171962

**Published:** 2023-03-06

**Authors:** Luiz R. O. Tosi, Paul William Denny, Camila I. De Oliveira, Jeziel D. Damasceno

**Affiliations:** ^1^ Department of Celular and Molecular Biology, Ribeirão Preto Medical School, University of São Paulo, São Paulo, Brazil; ^2^ Department of Biosciences, Durham University, Durham, United Kingdom; ^3^ Institute Gonçalo Muniz, Fiocruz, Salvador, Brazil; ^4^ The Wellcome Centre for Integrative Parasitology, Institute of Infection, Immunity and Inflammation, University of Glasgow, Glasgow, United Kingdom

**Keywords:** *Leishmania*, leishmaniasis, genome variability, genome plasticity, genome evolution

*Leishmania* are protozoan pathogens that cause leishmaniasis, a neglected disease with debilitating and potentially life-threatening symptoms. The *Leishmania* genome is very dynamic, with variations in both content and structure commonly reported. Typically, such heightened variation (i.e., genome plasticity), including chromosome and gene copy number variations, aneuploidy, and genome rearrangements, is associated with DNA instability in other organisms, Yet, in *Leishmania*, this inherent instability may be exploited, not only to introduce genome heterogeneity, but to modulate gene expression and generate fitness enhancing traits. We lack a clear and concise understanding of how these parasites regulate both plasticity and its potential consequences. Thus, the aim of this Research Topic was to collate significant reports investigating this ability of *Leishmania* to harness genome variability to their benefit, and to gather noteworthy reports on how genome plasticity impacts upon the clinical management of leishmaniasis and complications of an inherently unstable genome on our ability to genetic manipulate and study these unorthodox pathogens.

Copy number variations can alter gene dosage and, in *Leishmania*, these changes are believed to facilitate phenotypic plasticity within parasite populations. In this Research Topic, Valdivia et al. report extensive genome variability among parasite isolates from a leishmaniasis endemic region in Brazil, describing the rapid replacement of one dominant *L. infantum* karyotype within the population by a distinct subpopulation over a short 2 year period. Whether (and what) environmental factors could have driven such an expansion of one genotype relative to another within the population is still unknown. However, the diversity of genotypes retained amongst these isolates could hint at a selectable pool of alternative genomes that can be rapidly expanded in response to external stimuli. That said, despite frequent reports of CNVs in *Leishmania*, the impacts and biological consequences of these variations on gene expression is also important to consider, given *Leishmania* appear to lack conventional mechanisms for gene expression control. Herein, Silva et al. tested for such associations between transcriptomic and proteomic data across different life cycle stages of several *Leishmania* species, observing for a significant number of factors, a lack of correlation between transcriptomic and proteomic changes. Thus, these findings indicate that transcript levels should be carefully considered when characterizing *Leishmania* phenotypes.

A more recent emergence in the field of *Leishmania* gene expression regulation is the potential for epistatic interactions to influence phenotypic variability. Although definitive evidence that epistasis modulates genome expression in this parasite is limited, Alpizar-Sosa et al. describe a potential, functional interaction between two genes; when a putative sterol transporter (ABC3A) is lost, it is possible to knockout the catalytic subunit of serine palmitoyl transferase (LCB2), an otherwise essential gene in these parasites. Interestingly, LCB2 was initially deemed non-essential as LCB2-null mutants were readily selected using conventional KO approaches. However, whole genome sequencing revealed that selected LCB2-null mutants also lacked the ABC3A transporter gene. Moreover, this putative epistatic interaction was further supported when LCB2 and ABC3A knockouts were reproduced using CRISPR-Cas9 editing technology. Indeed, the introduction of CRISPR/Cas9 genome editing in *Leishmania* has significantly accelerated functional genomics analysis throughout the past decade. Until recently however, species such as *L. braziliensis* remained challenging to genetically manipulate. In this issue, Espada et al. report the adaption of the CRISPR system, originally derived from *L. mexicana*, and demonstrated its efficiency for editing genes in *L. braziliensis*. Their work provides a valuable tool for the study of this causative agent of tegumentary leishmaniasis in South America.

Another key driver of genome evolution is DNA instability; however, instability must be limited to prevent genome collapse. Tackling DNA instability in eukaryotes is the DNA Damage Response (DDR), a collection of pathways that evolved to detect and resolve erogenous DNA. ATM and ATR are kinases at the DDR apex, orchestrating fundamental aspects of genome stability, such as responses to specific types of DNA lesions or impaired DNA replication. In this Research Topic, Silva et al. investigated the effects of ATM and ATR inhibition in *Leishmania*, uncovering a role for these kinases in regulating the levels of single-stranded DNA under genotoxic conditions, suggesting these enzymes play important roles in *Leishmania* DNA stability. Indeed, the “druggability” of kinases makes these enzymes enticing targets for future studies, and perhaps offers hope for novel anti-leishmanial chemotherapy. Treatment of leishmaniasis is limited by a challenging disease management programme which relies upon a small arsenal of drugs that have considerable toxicity. Furthermore, the rise in drug resistant *Leishmania* populations threatens the potential for leishmaniasis elimination. Such concerns are raised in this Research Topic by Santi and Murta. Their review both discusses and summarizes different aspects of *Leishmani*a genome plasticity that could underlie mechanism of resistance to the main drugs used in anti-leishmanial chemotherapy, such as antimonials, miltefosine and amphotericin B. These authors also share their impressions on the impact of genome variability on the development of effective therapeutic tools to counter this neglected disease.

Adequate treatment and control of leishmaniasis is also heavily dependent on efficient strategies to identify and discriminate amongst different species of *Leishmania* to provide effective treatments. Diagnosis of cutaneous leishmaniasis using DNA-based approaches can be challenging, however some PCR-based strategies have proven to be highly specific and sensitive for the identification and discrimination of *Leishmania* species from patient samples. Fotouhi-Ardakani et al. contribution to this Research Topic through their discussion of the difficulties in diagnosing *Leishmania* infection and describes the development of TaqMan probe-based qPCR assays for specific detection of *L. major* and *L. tropica* in endemic areas in Iran.

In conclusion, the papers in this Research Topic add novel insights and perspectives to key aspects of *Leishmania* biology, bringing together diverse examples of where *Leishmania* genome variability governs not only the parasite homeostasis and evolution, but also the management of leishmaniasis and our ability to study this unusual pathogen.

## Author contributions

All authors listed have made a substantial, direct, and intellectual contribution to the work and approved it for publication.

